# Measuring the neighborhood environment: associations with young girls' energy intake and expenditure in a cross-sectional study

**DOI:** 10.1186/1479-5868-7-52

**Published:** 2010-06-01

**Authors:** Cindy W Leung, Steven E Gregorich, Barbara A Laraia, Lawrence H Kushi, Irene H Yen

**Affiliations:** 1 Departments of Nutrition and Epidemiology, Harvard School of Public Health, 665 Huntington Avenue, Boston, MA 02215 USA; 2 Division of General Internal Medicine, Department of Medicine, University of California, San Francisco, 3333 California Street, Suite 335, Box 0856, San Francisco, CA 94143-0856 USA; 3 Division of Prevention Sciences, Department of Medicine, University of California, San Francisco, 3333 California Street, Suite 465, San Francisco, CA 94118 USA; 4 Division of Research, Kaiser Permanente, 2000 Broadway, Oakland, CA 94612 USA

## Abstract

**Background:**

Neighborhood environments affect children's health outcomes. Observational methods used to assess neighborhoods can be categorized as indirect, intermediate, or direct. Direct methods, involving in-person audits of the neighborhoods conducted by trained observers, are recognized as an accurate representation of current neighborhood conditions. The authors investigated the associations of various neighborhood characteristics with young girls' diet and physical activity.

**Methods:**

This study is based on a subset of participants in the Cohort Study of Young Girls' Nutrition, Environment and Transitions (CYGNET). In-person street audits were conducted within 215 girls' residential neighborhoods using a modified St. Louis Audit Tool. From the street audit data, exploratory factor analysis revealed five neighborhood scales: "mixed residential and commercial," "food and retail," "recreation," "walkability," and "physical disorder." A Neighborhood Deprivation Index was also derived from census data. The authors investigated if the five neighborhood scales and the Neighborhood Deprivation Index were associated with quartiles of total energy intake and expenditure (metabolic equivalent (MET) hours/week) at baseline, and whether any of these associations were modified by race/ethnicity.

**Results:**

After adjustment for demographic characteristics, there was an inverse association between prevalence of "food and retail" destinations and total energy intake (for a one quartile increase, OR = 0.84, 95% CI 0.74, 0.96). Positive associations were also observed between the "recreation" and "walkability" scales with physical activity among Hispanic/Latina girls (for a one quartile increase in MET, OR = 1.94, 95% CI 1.31, 2.88 for recreation; OR = 1.71, 95% CI 1.11, 2.63 for walkability). Among African-American girls, there was an inverse association between "physical disorder" and physical activity (OR = 0.31, 95% CI 0.12, 0.80).

**Conclusions:**

These results suggest that neighborhood food and retail availability may be inversely associated with young girls' energy intakes in contrast to other studies' findings that focused on adults. There is considerable variation in neighborhoods' influences on young girls' physical activity behaviors, particularly for young girls of different racial/ethnic backgrounds.

## Background

Neighborhoods are geographic and social units that can have profound effects on health outcomes. For children, neighborhoods can encompass the schools they attend, the grocery stores their families patronize, the streets and roadways they travel on, and the other children and adults with whom they interact. These components also help to define different sub-neighborhood environments, such as the physical activity environment, the consumer environment, or the "information environment," [1] each of which may have different effects on children's health.

Previous studies have shown that neighborhood characteristics are associated with children's health outcomes, including low birth weight [2-4], childhood lead poisoning [5], asthma [6-9], pedestrian fatalities [10], and cognitive development [11,12]. As rates of childhood obesity have increased, researchers have focused on how certain neighborhood characteristics may affect children's diet and physical activity.

Observational methods used to assess the neighborhood environment can be indirect, intermediate, and direct [13]. Indirect methods involve combining census or geographic information systems data to create indices of neighborhood/area deprivation, such as the Neighborhood Deprivation Index [14], Townsend Material Development Score [15], or the Carstairs Deprivation Index [16]. Intermediate measures include using aerial photography or telephone book yellow pages to determine regional land-use and neighborhood composition [17]. Direct methods involve in-person audits of the neighborhood conducted by trained observers. Direct methods may capture elements missing from indirect or intermediate methods, such as sidewalk width or landscape maintenance [18], and unlike other methods, are not limited by databases that can have out-of-date information [13].

Davison and Lawson [19] reviewed 33 studies about neighborhood environments and children's physical activity. Access to recreational facilities and schools, presence of sidewalks, access to destinations and public transportation, were each associated with increased physical activity in children. Conversely, higher crime rates, physical disorder (e.g. presence of graffiti or empty beer bottles) and area deprivation (e.g. rates of car ownership, unemployment and crowding) were all negatively associated with children's physical activity. Neighborhood effects on children's diet have been less pronounced. Burdette and Whitaker [20] found no association between proximity to fast food restaurants and childhood obesity. Other evidence [21-23] has shown that school-based interventions can promote children's consumption of fruits and vegetables. However, most of the previously mentioned studies have used indirect or intermediate measures of the neighborhood environment. Few studies have examined the association between neighborhood characteristics and total energy intake, especially among younger children.

In addition, race/ethnicity may act as an effect modifier in the associations between the neighborhood environment and total energy intake and expenditure. Previous studies have already reported disparities in the local food environment. Individuals residing in neighborhoods with predominantly White residents generally have more access to chain supermarkets, fruit and vegetable markets, and bakeries than individuals residing in neighborhoods with predominantly low-income or minority residents [24,25]. A study from the Youth Risk Behavior Survey [26] found that living in neighborhoods with high proportions of Hispanic residents was associated with more healthful dietary habits while living in low socioeconomic neighborhoods was associated with poorer dietary habits among adolescents. Another study of eighth-grade girls in South Carolina [27], found that White girls reported greater access to sports equipment, ability to walk safely in the neighborhood, and self-efficacy for physical activity than African-American girls.

In this study, we examined associations between neighborhood characteristics and energy intake and expenditure among young girls. We conducted in-person audits of the geographic neighborhoods in which these young girls resided, and used a factor analysis approach with these audit data to develop neighborhood scales. These scales and a combined Neighborhood Deprivation Index derived from census data were examined in association with measures of total energy intake and energy expenditure. Lastly, we examined whether these associations were modified by race/ethnicity.

## Materials and methods

### Study participants

This study is based on a subset of participants in the Cohort Study of Young Girls' Nutrition, Environment, and Transitions (CYGNET). A project of the Bay Area Breast Cancer and the Environment Research Center, the CYGNET Study is a prospective cohort study that focuses on identifying environmental and other exposures associated with differences in age-at-onset of pubertal development [28]. The CYGNET Study began in 2005 when 444 girls were recruited from the Kaiser Permanente Northern California (KPNC) health plan membership. Participants for the CYGNET Study were identified through the KPNC Infant Cohort File, a database containing information on all live births occurring in KPNC facilities. Children were eligible for recruitment if they were female, age six or seven years at time of recruitment, current members of KPNC, residents in Marin County, San Francisco, and select East Bay Communities currently and at the time of birth, and not intending to move from the area in the near future. Exclusion criteria included having a preexisting medical condition known to influence puberty or a psychiatric condition that could potentially limit study participation.

From June 2005 to August 2006, participants completed a baseline clinical visit where a range of clinical assessments were conducted, including collection of blood and urine samples, anthropometric measurements, and Tanner stage evaluation. Tanner stage evaluation consists of five stages of breast and pubic hair development, and is used to assess the degree of pubertal development of young girls [29]. The visit also included an interview with the primary caregiver about chemical exposures, weight changes, and residential history. Participants were informed that they would be called approximately every three months to assess food intake by a telephone 24-hour dietary recall. Dietary recalls were conducted by trained interviewers at the Cincinnati Children's Hospital Dietary Data Entry Center, using methods developed by the University of Minnesota Nutrition Coordinating Center. All CYGNET Study procedures were approved by the Kaiser Permanente Institutional Review Board.

### Study procedures and data collection

From the CYGNET study, 215 girls were randomly selected to participate in an auxiliary study on the relation between neighborhood environments and dietary and physical activity behaviors. Direct observations of the girls' neighborhoods were conducted using a modified version of the St. Louis Audit Tool [30], which consisted of a 68-item checklist of mostly dichotomous response categories (e.g. visible or not visible). Audit tool items fit into five categories: land use environment, transportation environment, facilities, park or playground contents, and physical disorder.

**Figure 1:**
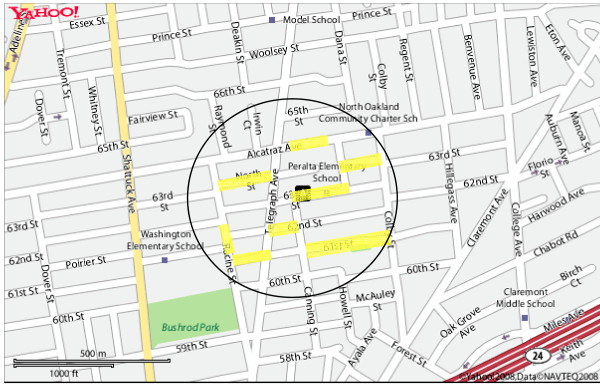
**Sample map of a quarter-mile radius around an arbitrary residence demonstrating the highlighted streets audited by street observers**.

Street observers were provided a map with a circle representing a quarter-mile radius drawn around each girl's residence. In order to preserve participant confidentiality, we have provided a sample map using an arbitrary street address to illustrate the selections of streets audited by street observers (Figure 1). Random selections of every third street segment were highlighted inside the circle. A street segment was defined as the length of the street to the nearest crossroad or major curve. Partial street segments were excluded from observation. The street observers completed one audit form for both sides of each highlighted street segment and were blinded to the addresses of the girls' residences. The range of street segments observed per girl ranged from 1 to 32 segments, depending on the length and density of the segments within each neighborhood. A mean of 10.8 segments were observed per girl, with 2,328 street segments observed in total. To ensure accuracy, duplicate audits were conducted on 246 street segments by different observers. Thirty total discrepancies were found among 16,728 audit tool observations, yielding a 0.2% disagreement rate. The physical disorder category (i.e. whole or broken bottles, garbage or litter) contributed the majority of discrepancies noted between observers.

Neighborhood data were collected from June to November of 2007. The auxiliary study was approved by both UCSF and Kaiser Permanente's Institutional Review Boards.

### Dependent variables

Dietary and physical activity variables were collected as part of the CYGNET Study.

#### Diet

Total energy intake was calculated from 24-hour dietary recalls collected up to four times over the course of a year and approximately three months apart. The girl and her parent or guardian most knowledgeable of the girl's diet completed the 24-hour recalls. Total energy intake was averaged over all 24-hour recalls completed in the baseline year. Girls who had completed only one 24-hour recall were excluded (n = 3). For analysis, total energy intake was categorized by quartiles with cut-points at 1365.30, 1554.19, and 1745.54 kilocalories.

#### Physical activity

Physical activity was measured in metabolic equivalents (MET), where one MET is defined as the energy requirement per kilogram of body weight per hour of activity divided by the energy requirement per kilogram of body weight at rest [31]. Data were based on a physical activity questionnaire that collected information about the number of hours per week over a one-year period that a girl participated in various physical activities, such as dance, soccer, basketball, and swimming, in the baseline year. Individual activities were converted to MET values, which contributed to each girl's total energy expenditure. Questions from the physical activity questionnaire have been previously validated in other studies [32]. For each participant, responses for these activities were combined to derive an estimate of total MET hours per week. Girls who had incomplete or missing MET hours per week were excluded (n = 6). Because the data were positively skewed, physical activity was categorized into quartiles with cut-points at 1.20, 8.33, and 16.00 MET hours per week for analysis.

### Neighborhood measures

#### Neighborhood Deprivation Index

The Neighborhood Deprivation Index was calculated using the method described by Messer et al. [14]. The Index is an empirical summary of socioeconomic deprivation based on eight census variables: % of individuals with income in 1999 below poverty level; % of families with female headed households with no husband present and children under age 18 y; % of households with incomes under $30,000/year; % of households with public assistance income; % of people age 16 or older in civilian labor force currently unemployed; % of males in management; % of all persons age 25 or older with less than a high school degree; and, % of households with more than one person per room. Using census data, we fit a principal component analysis (PCA) to obtain the item loadings, which were used to weight each census variable's contribution to the first principal component. The component was standardized to create individual Neighborhood Deprivation Indices for census tracts and counties within the study area.

#### Neighborhood scales

Neighborhood audit data were averaged across the number of street segments observed per girl, in order to preserve the unit of observation as the individual. Because of the skewed distribution of several neighborhood items, we did not transform the neighborhood items as this would not have helped to normalize the distributions. Rather, the resulting means were dichotomized with the value 1 if the targeted attribute was visible on any observed street segment within a quarter-mile radius of the girl's residence, and 0 if not visible.

#### Other covariates

Weight was measured in kilograms, using a calibrated digital Tanita^® ^scale TBF 300A, where participants stood without shoes or socks. Height was measured in centimeters using a fixed stadiometer. Body mass index (BMI) was calculated as the ratio of weight in kilograms over the square of height in meters. BMI-for-age ≥85^th ^percentile, using age (in months)-specific distribution standards from the Centers for Disease Control and Prevention, was defined as overweight and obese.

Covariates for multivariable regression analyses included the girl's age at time of clinic visit (to the nearest tenth year), race/ethnicity (Non-Hispanic White, African-American, Asian, Hispanic/Latina, "Other or Multiple"), BMI, household size, age of birth mother, and household income (< $25,000; $25,000-$49,999; $50,000-$74,999; $75,000-$99,999; ≥$100,000).

### Statistical analysis

We estimated the tetrachoric correlation matrix of the street audit items and fit an exploratory factor analysis (EFA) with oblique rotation [33], to reduce the dimensionality of the neighborhood audit items. We chose EFA in order to identify the underlying variables contributing to the shared variance of the observed neighborhood items without *a priori *hypotheses of how these relationships may exist. Prior to EFA analysis, specific neighborhood item variables were removed due to insufficient variation in their distributions across the study population. Multiple EFA models were fit to obtain the number of factors with the "cleanest" structure [34]. Neighborhood item variables were removed individually if they failed to load ≥0.45 on any factor or if they had similar loadings on multiple factors. Forty neighborhood item variables were retained in the final model. We then created neighborhood scales by summing the responses of the corresponding items. The internal reliability of the neighborhood scales was assessed by calculating Cronbach's alphas.

The relationships between the Neighborhood Deprivation Index and the neighborhood scales derived from EFA were estimated by Pearson correlation coefficients. Two girls were excluded from the analytical sample due to missing Neighborhood Deprivation Indices.

Next, using ordinal logistic models, total energy intake quartiles and physical activity quartiles were regressed onto the neighborhood measures. The Neighborhood Deprivation Index and neighborhood scales were introduced separately into the model. The first model adjusted for the total number of street segments observed per girl. The second model included age, race/ethnicity, and BMI. The third model added covariates of household size, age of birth mother and household income. Girls were excluded from the third model if their household income was missing (n = 3). Fitted models were single-level models because girls were originally sampled from a singular database based on being members of KPNC at time of birth, rather than sampled from a geographic entity (i.e. a census block).

Last, we assessed whether weight status or race/ethnicity modified the associations between neighborhood measures and total energy intake and physical activity. Interaction terms corresponding to each neighborhood measure were introduced into the third model. Statistical tests were two-sided and significance was determined at *P *< 0.05. All statistical analyses were performed using SAS 9.2 (SAS Institute Inc., Cary, NC, USA).

## Results

Baseline sociodemographic characteristics of the study population are shown in Table 1. The mean age was 7.4 (0.4) years. The sample was comprised of mostly non-Hispanic Whites (40.5%), followed by Hispanics/Latinas (23.3%), and African-Americans (19.1%). Mean BMI was 17.3 (3.0) kg/m^2^, and 29.8% of girls were overweight or obese, as defined by having a BMI-for-age ≥85^th ^percentile.

**Table 1:** Baseline Sociodemographic Characteristics of Study Participants (n = 215)

	N	%
		
Age, mean (SD)	7.4 (0.4)	Range: 6.5-8.1
Age of birth mother, mean (SD)	40.0 (5.8)	Range: 22.0-54.0
Height in cm, mean (SD)	125.0 (6.2)	Range 114.0-144.5
Weight in kg, mean (SD)	27.2 (6.8)	Range 16.8-57.3
Body mass index (BMI), mean (SD)	17.3 (3.0)	Range 12.2, 29.0
Household size, mean (SD)	4.1 (1.0)	Range 2.0-8.0
Total energy intake, kcals, mean (SD)	1572.9 (316.8)	Range 810.4 - 2760.3
Physical activity, MET hours/week, mean (SD)	11.9 (14.3)	Range 0 - 80.0
		
Race/Ethnicity		
Non-Hispanic White	87	40.5
African-American	41	19.1
Asian	10	4.7
Hispanic/Latina	50	23.3
Other or Multiple	27	12.6
Overweight status (BMI-for-age ≥85th percentile)		
Not overweight	151	70.2
Overweight or obese	64	29.8
Household income		
< $25,000	11	5.1
$25,000 - $49,999	34	15.8
$50,000 - $74,999	33	15.3
$75,000 - $99,999	43	20.0
≥ $100,000	91	42.3
Missing	3	1.4

MET, metabolic equivalent

Six scales initially emerged from the EFA on the neighborhood items (see additional file 1: Neighborhood Audit Tool Items, Standardized Regression Coefficients and Raw Cronbach's Alphas for Scales Derived From Exploratory Factor Analysis). The first scale, which we called "mixed residential and commercial," (Cronbach's α = 0.87) was associated with 14 items, including residential properties, convenience stores, and places of worship. The second scale, called "food and retail" (α = 0.83), was comprised of 11 items, including restaurants, supermarkets, and commercial destinations. The third scale, called "recreation" (α = 0.76) was comprised of parks, playgrounds, sports/playing fields, walking trails, and "complete sports equipment." The fourth scale called "walkability" (α = 0.50) was comprised of street shoulders, curb extensions, traffic circles, street signs asking drivers to watch out for children, and playground equipment. The fifth scale, called "physical disorder" (α = 0.58) was comprised of garbage, litter or broken glass; graffiti; railroad tracks, bridge, tunnel, highway or overpass; miscellaneous government buildings; and coffee shops. Only one item, "incomplete sports equipment," loaded on a sixth scale; therefore, we eliminated this scale from subsequent analyses.

The correlations between the five neighborhood scales derived from EFA and the Neighborhood Deprivation Index derived from census information are shown in Table 2. The Neighborhood Deprivation Index was modestly positively correlated with both the "mixed residential and commercial" and "physical disorder" scales. The "mixed residential and commercial" scale was also positively correlated with "food and retail" and "physical disorder."

**Table 2:** Correlation between neighborhood deprivation and neighborhood scales (n = 213)^a^

	Neighborhood Deprivation Index	Scales derived from Exploratory Factor Analysis				
		
		Mixed residential and commercial	Food and retail	Recreation	Walkability	Physical disorder
Neighborhood deprivation score	1.00					
Mixed residential and commercial	0.51**	1.00				
Food and retail	0.08	0.63**	1.00			
Recreation	-0.04	0.19*	0.15*	1.00		
Walkability	0.12	0.37**	0.30**	0.56**	1.00	
Physical disorder	0.33**	0.65**	0.57**	0.14*	0.33**	1.00

* P > 0.05

** P > 0.0001

^a ^Two girls were excluded from regression analyses due to missing Neighborhood Deprivation Index

Table 3 presents the results of logistic regression analyses for total energy intake. After adjusting for all covariates, "food and retail" was inversely related to total energy intake, where each one-item increase on the "food and retail" scale was associated with a 16% decreased odds of consuming the next highest quartile of total energy (OR = 0.84, 95% CI 0.74, 0.96). Race/ethnicity did not modify the associations between neighborhood measures and total energy intake.

**Table 3:** Associations Between the Neighborhood Environment and Total Energy Intake (kcals/day) (n = 210)^a^

Model description	Neighborhood Deprivation Index		Scales derived from Exploratory Factor Analysis									
			
			Mixed residential and commercial		Food and retail		Recreation		Walkability		Physical disorder	
	
	OR	95% CI	OR	95% CI	OR	95% CI	OR	95% CI	OR	95% CI	OR	95% CI
Model 1^b^	0.97	0.88, 1.07	0.94	0.86, 1.03	0.83*	0.73, 0.94	1.02	0.87, 1.21	1.12	0.91, 1.37	0.92	0.70, 1.20
Model 2^c^	0.97	0.85, 1.10	0.94	0.85, 1.03	0.84*	0.74, 0.95	1.03	0.87, 1.22	1.06	0.86, 1.32	0.97	0.73, 1.29
Model 3^d^	0.98	0.85, 1.12	0.93	0.84, 1.03	0.84*	0.74, 0.96	0.98	0.82, 1.17	1.01	0.81, 1.26	0.95	0.71, 1.27

* P > 0.05

^a ^Five girls were excluded from regression analyses due to missing Neighborhood Deprivation Index or total caloric intake.

^b ^Adjusted for total number of streets observed per girl.

^c ^Adjusted for total number of streets observed per girl, age, race/ethnicity, and BMI.

^d ^Adjusted for total number of streets observed per girl, age, race/ethnicity, BMI, household size, age of birth mother, and household income. Three girls were further excluded due to missing household income information. N = 207

The relations between neighborhood measures and physical activity, as measured in MET hours per week, are shown in Table 4. In the first model, adjusting for total number of street segments observed per girl, there were significant inverse associations between physical activity and neighborhood deprivation (OR = 0.80, 95% CI 0.72, 0.89), "mixed residential and commercial" destinations (OR = 0.91, 95% CI 0.83, 0.99), and "physical disorder" (OR = 0.73, 95% CI 0.55, 0.96). However, these associations were attenuated after adjusting for other covariates.

**Table 4:** Associations Between the Neighborhood Environment and Physical Activity (MET hours/week) (n = 207)^a^

Model description	Neighborhood Deprivation Index		Scales derived from Exploratory Factor Analysis									
			
			Mixed residential and commercial		Food and retail		Recreation		Walkability		Physical disorder	
	
	OR	95% CI	OR	95% CI	OR	95% CI	OR	95% CI	OR	95% CI	OR	95% CI
Model 1^b^	0.80*	0.72, 0.89	0.91*	0.83, 0.99	1.03	0.91, 1.16	1.14	0.96, 1.36	1.11	0.91, 1.37	0.73*	0.55, 0.96
Model 2^c^	0.89	0.78, 1.01	0.96	0.87, 1.06	1.00	0.88, 1.13	1.10	0.92, 1.31	1.08	0.87, 1.34	0.78	0.58, 1.04
Model 3^d^	0.92	0.80, 1.06	0.99	0.89, 1.10	1.01	0.88, 1.15	1.10	0.91, 1.32	1.09	0.86, 1.37	0.80	0.59, 1.07
*Interaction with race/ethnicity^d^*												
Non-Hispanic White	1.30	0.91, 1.86	0.97	0.83, 1.13	0.98	0.80, 1.20	1.01	0.75, 1.34	0.97	0.72, 1.30	0.92	0.63, 1.34
African-American	0.86	0.68, 1.09	0.99	0.82, 1.18	0.81	0.57, 1.14	0.77	0.47, 1.26	0.91	0.50, 1.64	0.31*	0.12, 0.80
Asian	0.43	0.09, 2.12	0.88	0.59, 1.30	1.12	0.75, 1.68	0.90	0.44, 1.84	0.21	0.03, 1.55	1.46	0.03, 69.37
Hispanic/Latina	0.79	0.63, 1.01	0.98	0.83, 1.14	0.95	0.75, 1.21	1.94*	1.31, 2.88	1.71*	1.11, 2.63	0.64	0.34, 1.20
Other or Multiple	1.12	0.76, 1.64	1.08	0.88, 1.31	1.10	0.87, 1.39	0.79	0.48, 1.30	0.95	0.49, 1.85	0.78	0.39, 1.56

MET, metabolic equivalent

* P < 0.05

^a ^Eight girls were excluded from regression analyses due to missing Neighborhood Deprivation Index or MET hours per week

^b ^Adjusted for total number of streets observed per girl.

^c ^Adjusted for total number of streets observed per girl, age, race/ethnicity, and BMI.

^d ^Adjusted for total number of streets observed per girl, age, race/ethnicity, BMI, household size, age of birth mother, and household income. Two girls were further excluded due to missing household income information. N = 205.

Some associations between the neighborhood measures and physical activity were modified by race/ethnicity. In our sample, Asian girls were most active, averaging 16.9 MET hours per week, and Hispanic/Latina girls were least active, averaging 9.0 MET hours per week. Among Hispanic/Latina girls, both neighborhood recreation and walkability were positively associated with physical activity. Each one-item increase on the "recreation" scale was associated with a 94% increased odds of engaging in the next highest quartile of physical activity (OR = 1.94, 95% CI 1.31, 2.88). Similarly, each one-item increase on the "walkability" scale was associated with a 71% increased odds of engaging in the next highest quartile of physical activity (OR = 1.71, 95% CI 1.11, 2.63). An inverse association was also observed between "physical disorder" and physical activity among African-American girls (OR = 0.31, 95% CI 0.12, 0.80).

## Discussion

This study examines the relations between neighborhood measures with total energy intake and expenditure among a sample of young girls, using both indirect and direct assessments of the neighborhood environment.

While there is considerable evidence for the associations between neighborhood characteristics and health outcomes among adults, these findings are not necessarily generalizeable to children. For adults, the literature suggests that living closer to supermarkets may increase fruit and vegetable intake [35,36], whereas living closer to fast-food restaurants is associated with obesity risk [37,38]. Contrary to these findings, we observed an inverse association between the prevalence of "food and retail" destinations and total energy intake among young girls. Compared to adults, children may be less influenced by environmental food availability because children usually have limited ability to purchase food without supervision. Fisher and Birch [39] found that restricting food access among young children promoted overeating when the restrictions were lifted. On the other hand, perhaps diversification of the neighborhood food environment deters energy consumption among children due to the unrestricted accessibility to assorted food options.

Our findings show crude inverse associations between physical activity and neighborhood deprivation, access to "mixed residential and commercial" destinations, and "physical disorder." These corroborate previous studies which found similar negative associations between children's physical activity and neighborhood violent crime [40] and physical and social disorder [41].

The significant interaction effects of race/ethnicity indicate that certain neighborhood characteristics are more strongly associated with physical activity among children of different racial/ethnic groups. For example, Hispanic/Latina girls' physical activity behaviors were positively associated with neighborhood "recreation" and "walkability." "Physical disorder" was also associated with lower physical activity behaviors among African-American girls. This may result from wider variation in these scales among different racial/ethnic groups, enhancing the ability to detect associations.

A considerable strength of this study was the use of street audit data, a method generally recognized as an accurate representation of current neighborhood conditions. By using EFA, we were able to use the underlying correlation pattern of numerous street items to develop our neighborhood scales. However, the EFA model is provisional and we encourage attempts at replication. The literature for the relationship between neighborhoods and children's dietary intake is still growing; our study suggests that neighborhood food and retail availability may influence children's dietary behaviors differently from that of adults. In contrast, the associations we observed between neighborhood measures and children's physical activity behaviors corroborate previous studies using different methods of neighborhood assessment.

One limitation of this study is its cross-sectional nature, which makes it difficult to infer causation. While most neighborhood studies also employ cross-sectional designs, longitudinal studies may better capture the fluidity of the neighborhood environment and how changes over time affect the health of its residents. On the other hand, many neighborhood aspects are fairly stable within the time frame of a few years, although changes in residence would also influence neighborhood characteristics.

Second, our sample size is relatively small compared with other studies. Davison and Lawson [19] observed that significant associations between the neighborhood environment and children's physical activity behaviors were found mostly in studies with 1,000 participants or more, suggesting that these associations are small and only detectable within large sample sizes. While the sample size limited our ability to validate the neighborhood scales, other studies have found similar associations between the neighborhood environment and children's physical activity using indirect neighborhood assessment methods. Investigators who are conducting direct observations could use these new scales to guide their data collection.

Estimates of energy expenditure were derived from girls' individual activities; however, values based on MET equivalents for adults' activities were used to estimate total energy expenditure among girls since MET equivalents for children's activities were not available. Because all girls were classified the same way, nondifferential misclassification would not affect the overall rankings of girl's energy expenditures relative to one another, though our effect estimates for energy expenditure might be attenuated. Estimates of energy intake and expenditure were derived from self- and maternal-reported data. Though under-reporting of dietary intake is possible, study dieticians used the multiple pass dietary assessment and collected several 24-hour recalls to improve energy intake estimates. It is unlikely that systematic measurement error would occur in self-reported food intake or physical activity patterns by neighborhood characteristics; therefore, crude associations may be attenuated but not otherwise biased.

There may be unmeasured confounding by parental behaviors and practices. Under the assumption that parents can select into more desirable communities (i.e. communities with low crime rates, good public schools, parks and recreational spaces), parental practices may be a common cause of the neighborhood environment and girls' energy intake and expenditure. Though we adjusted for parental income in our analyses, residual confounding may still exist. Future studies examining associations between the neighborhood environment and children's health behaviors should collect information on parental perceptions of the neighborhood to better control for any potential confounding.

The participants from the CYGNET Study may not be representative of the general population. All participants were members of KPNC at birth and at time of recruitment, although their health coverage in the interim has not been examined. The study sample also tended to be from higher socioeconomic status households. On the other hand, study participants are diverse racially and ethnically, with over 25 percent being Hispanic/Latina, reflecting the diversity of the San Francisco Bay Area. While non-representativeness suggests caution in extrapolating inferences to the general population, the aim of this study was to determine whether variations in neighborhood measures are associated with variations in energy intake or expenditure. As indicated by the substantial variation in the number of street segments that were observed per girl, we successfully achieved variation in the neighborhood measures.

Because the study population was comprised of young girls, we cannot generalize our findings to boys. Previous studies that have examined the neighborhood environment in relation to energy expenditure have been inconsistent, with some studies showing stronger associations in girls [42,43], and others showing larger effects among boys [44,45]. Further research is warranted to determine if neighborhood environments affect energy intake and expenditure differentially in girls and boys. Like other neighborhood studies, we also did not measure how participants used their neighborhood environments, nor did we measure the school environment. Therefore, we cannot determine if the observed associations between neighborhood measures and dietary and physical activity behaviors are direct or resultant from unmeasured characteristics. However, such information is now being collected in follow-up interviews with CYGNET Study participants, enabling future investigation of these aspects with the neighborhood measures presented here.

## Conclusion

Principal findings of this study include an inverse association between prevalence of "food and retail" destinations and total energy intake; positive associations of "recreation" and "walkability" with energy expenditure among Hispanic/Latina girls; and an inverse association of "physical disorder" with energy expenditure among African-American girls. These results suggest that neighborhood food and retail availability may be inversely associated with children's energy intakes in contrast to other studies' findings, which focused on adults. There is considerable variation in neighborhoods' influences on children's physical activity behaviors, particularly for children of different racial/ethnic backgrounds.

The current body of literature suggests that neighborhood characteristics can affect children's health outcomes through numerous processes. It is only when these mechanisms are better understood by health professionals, policymakers and community members that we can develop appropriate policy and behavioral changes to preserve children's health.

## Competing interests

The authors declare that they have no competing interests.

## Authors' contributions

CL created the analysis plan, conducted the analysis, contributed to the interpretation of the data, and drafted the manuscript. SG helped to design the study's statistical methodology. BL obtained the census variables and derived the Neighborhood Deprivation Index using these measures. LK helped prepare the Materials and Methods section, and is the principal investigator of the CYGNET data. IY directed the collection of the street audit data, contributed to the overall study design, helped to direct the study's implementation, and contributed to the interpretation of the data. All authors also contributed to critically reviewing and revising the intellectual content of the manuscript, and have approved the final submitted manuscript.

## Supplementary Material

Additional file 1 **Neighborhood Audit Tool Items, Standardized Regression Coefficients, and Raw Cronbach's Alphas for Scales Derived From Exploratory Factor Analysis**. This file describes the results from exploratory factor analysis, including the individual neighborhood audit tool items, standardized regression coefficients, and raw Cronbach's alphas for each of the five derived scales. Click here for file

## References

[B1] Sallis JF Glanz K Physical activity and food environments: solutions to the obesity epidemic Milbank Q 2009 87 123 154 10.1111/j.1468-0009.2009.00550.x 19298418 PMC2879180

[B2] Buka SL Brennan RT Rich-Edwards JW Raudenbush SW Earls F Neighborhood support and the birth weight of urban infants Am J Epidemiol 2003 157 1 8 10.1093/aje/kwf170 12505884

[B3] Collins JW Jr Wambach J David RJ Rankin KM Women's lifelong exposure to neighborhood poverty and low birth weight: a population-based study Matern Child Health J 2009 13 326 333 10.1007/s10995-008-0354-0 18459039

[B4] English PB Kharrazi M Davies S Scalf R Waller L Neutra R Changes in the spatial pattern of low birth weight in a southern California county: the role of individual and neighborhood level factors Soc Sci Med 2003 56 2073 2088 10.1016/S0277-9536(02)00202-2 12697198

[B5] Sargent JD Brown MJ Freeman JL Bailey A Goodman D Freeman DH Jr Childhood lead poisoning in Massachusetts communities: its association with sociodemographic and housing characteristics Am J Public Health 1995 85 528 534 10.2105/AJPH.85.4.528 7702117 PMC1615119

[B6] Dales R Wheeler AJ Mahmud M Frescura AM Liu L The influence of neighborhood roadways on respiratory symptoms among elementary schoolchildren J Occup Environ Med 2009 51 654 660 10.1097/JOM.0b013e3181a0363c 19448574

[B7] Juhn YJ Sauver JS Katusic S Vargas D Weaver A Yunginger J The influence of neighborhood environment on the incidence of childhood asthma: a multilevel approach Soc Sci Med 2005 60 2453 2464 10.1016/j.socscimed.2004.11.034 15814171

[B8] Lovasi GS Quinn JW Neckerman KM Perzanowski MS Rundle A Children living in areas with more street trees have lower prevalence of asthma J Epidemiol Community Health 2008 62 647 649 10.1136/jech.2007.071894 18450765 PMC3415223

[B9] Newcomb P Li J Predicting admissions for childhood asthma based on proximity to major roadways J Nurs Scholarsh 2008 40 319 325 10.1111/j.1547-5069.2008.00245.x 19094146

[B10] Mueller BA Rivara FP Bergman AB Urban-rural location and the risk of dying in a pedestrian-vehicle collision J Trauma 1988 28 91 94 10.1097/00005373-198801000-00013 3339668

[B11] Caughy MO Hayslett-McCall KL O'Campo PJ No neighborhood is an island: incorporating distal neighborhood effects into multilevel studies of child developmental competence Health Place 2007 13 788 798 10.1016/j.healthplace.2007.01.006 17382574

[B12] Santos DN Assis AM Bastos AC Santos LM Santos CA Strina A Prado MS Almeida-Filho NM Rodrigues LC Barreto ML Determinants of cognitive function in childhood: a cohort study in a middle income context BMC Public Health 2008 8 202 10.1186/1471-2458-8-202 18534035 PMC2442073

[B13] Booth KM Pinkston MM Poston WS Obesity and the built environment J Am Diet Assoc 2005 105 S110 117 10.1016/j.jada.2005.02.045 15867906

[B14] Messer LC Laraia BA Kaufman JS Eyster J Holzman C Culhane J Elo I Burke JG O'Campo P The development of a standardized neighborhood deprivation index J Urban Health 2006 83 1041 1062 10.1007/s11524-006-9094-x 17031568 PMC3261293

[B15] Townsend PPP Beattie A Health and deprivation: inequality and the North 1988 London: Routledge

[B16] Carstairs V Morris R Deprivation and Health in Scotland 1990 Aberdeen University Press 2394583

[B17] Yen IH Kaplan GA Neighborhood social environment and risk of death: multilevel evidence from the Alameda County Study Am J Epidemiol 1999 149 898 907 10342798 10.1093/oxfordjournals.aje.a009733

[B18] Brownson RC Hoehner CM Day K Forsyth A Sallis JF Measuring the built environment for physical activity: state of the science Am J Prev Med 2009 36 S99 123 e112 10.1016/j.amepre.2009.01.005 19285216 PMC2844244

[B19] Davison KK Lawson CT Do attributes in the physical environment influence children's physical activity? A review of the literature Int J Behav Nutr Phys Act 2006 3 19 10.1186/1479-5868-3-19 16872543 PMC1557665

[B20] Burdette HL Whitaker RC Neighborhood playgrounds, fast food restaurants, and crime: relationships to overweight in low-income preschool children Prev Med 2004 38 57 63 10.1016/j.ypmed.2003.09.029 14672642

[B21] Blanchette L Brug J Determinants of fruit and vegetable consumption among 6-12-year-old children and effective interventions to increase consumption J Hum Nutr Diet 2005 18 431 443 10.1111/j.1365-277X.2005.00648.x 16351702

[B22] French SA Stables G Environmental interventions to promote vegetable and fruit consumption among youth in school settings Prev Med 2003 37 593 610 10.1016/j.ypmed.2003.09.007 14636793

[B23] Reynolds KD Franklin FA Binkley D Raczynski JM Harrington KF Kirk KA Person S Increasing the fruit and vegetable consumption of fourth-graders: results from the high 5 project Prev Med 2000 30 309 319 10.1006/pmed.1999.0630 10731460

[B24] Powell LM Slater S Mirtcheva D Bao Y Chaloupka FJ Food store availability and neighborhood characteristics in the United States Prev Med 2007 44 189 195 10.1016/j.ypmed.2006.08.008 16997358

[B25] Moore LV Diez Roux AV Associations of neighborhood characteristics with the location and type of food stores Am J Public Health 2006 96 325 331 10.2105/AJPH.2004.058040 16380567 PMC1470485

[B26] Lee RE Cubbin C Neighborhood context and youth cardiovascular health behaviors Am J Public Health 2002 92 428 436 10.2105/AJPH.92.3.428 11867325 PMC1447094

[B27] Felton GM Dowda M Ward D Dishman R Trost S Saunders R Pate R Differences in Physical Activity Between Black and White Girls Living in Rural and Urban Areas J Sch Health 2002 72 250 255 10.1111/j.1746-1561.2002.tb07338.x 12212410

[B28] Wolff MS Teitelbaum SL Pinney SM Windham G Liao L Biro F Kushi LH Erdmann C Hiatt RA Rybak ME Calafat AM Investigation of Relationships between Urinary Biomarkers of Phytoestrogens, Phthalates, and Phenols and Pubertal Stages in Girls Environ Health Perspect10.1289/ehp.0901690 PMC2920905 20308033

[B29] Bonat S Pathomvanich A Keil MF Field AE Yanovski JA Self-assessment of pubertal stage in overweight children Pediatrics 2002 110 743 747 10.1542/peds.110.4.743 12359788

[B30] Brownson RC Hoehner CM Brennan LK Cook RA Elliott MB McMullen KM Reliability of 2 Instruments for Auditing the Environment for Physical Activity Journal of Physical Activity and Health 2004 1 191 208

[B31] Hu FB Sigal RJ Rich-Edwards JW Colditz GA Solomon CG Willett WC Speizer FE Manson JE Walking compared with vigorous physical activity and risk of type 2 diabetes in women: a prospective study JAMA 1999 282 1433 1439 10.1001/jama.282.15.1433 10535433

[B32] Kimm SY Glynn NW Kriska AM Fitzgerald SL Aaron DJ Similo SL McMahon RP Barton BA Longitudinal changes in physical activity in a biracial cohort during adolescence Med Sci Sports Exerc 2000 32 1445 1454 10.1097/00005768-200008000-00013 10949011

[B33] Goldberg LR Digman JM Strack S, Lorr M Revealing structure in the data: Principles of exploratory factor analysis Differentiating normal and abnormal personality 1994 New York: Springer 216 242

[B34] Costello AB Osborne J Best practices in exploratory factor analysis: four recommendations for getting the most from your analysis Book Best practices in exploratory factor analysis: four recommendations for getting the most from your analysis. Editor ed.^eds 2005 10

[B35] Morland K Wing S Diez Roux A The contextual effect of the local food environment on residents' diets: the atherosclerosis risk in communities study Am J Public Health 2002 92 1761 1767 10.2105/AJPH.92.11.1761 12406805 PMC1447325

[B36] Zenk SN Lachance LL Schulz AJ Mentz G Kannan S Ridella W Neighborhood retail food environment and fruit and vegetable intake in a multiethnic urban population Am J Health Promot 2009 23 255 264 19288847 10.4278/ajhp.071204127 PMC3305995

[B37] Inagami S Cohen DA Brown AF Asch SM Body Mass Index, Neighborhood Fast Food and Restaurant Concentration, and Car Ownership J Urban Health 2009 19533365 10.1007/s11524-009-9379-y PMC2729867

[B38] Li F Harmer P Cardinal BJ Bosworth M Johnson-Shelton D Moore JM Acock A Vongjaturapat N Built environment and 1-year change in weight and waist circumference in middle-aged and older adults: Portland Neighborhood Environment and Health Study Am J Epidemiol 2009 169 401 408 10.1093/aje/kwn398 19153214 PMC2726645

[B39] Fisher JO Birch LL Restricting access to palatable foods affects children's behavioral response, food selection, and intake Am J Clin Nutr 1999 69 1264 1272 10357749 10.1093/ajcn/69.6.1264

[B40] Gomez JE Johnson BA Selva M Sallis JF Violent crime and outdoor physical activity among inner-city youth Prev Med 2004 39 876 881 10.1016/j.ypmed.2004.03.019 15475019

[B41] Molnar BE Gortmaker SL Bull FC Buka SL Unsafe to play? Neighborhood disorder and lack of safety predict reduced physical activity among urban children and adolescents Am J Health Promot 2004 18 378 386 15163139 10.4278/0890-1171-18.5.378

[B42] Mota J Gomes H Almeida M Ribeiro JC Santos MP Leisure time physical activity, screen time, social background, and environmental variables in adolescents Pediatr Exerc Sci 2007 19 279 290 18019587 10.1123/pes.19.3.279

[B43] Spence JC Cutumisu N Edwards J Evans J Influence of neighbourhood design and access to facilities on overweight among preschool children Int J Pediatr Obes 2008 3 109 116 10.1080/17477160701875007 18465437

[B44] Colabianchi N Kinsella AE Coulton CJ Moore SM Utilization and physical activity levels at renovated and unrenovated school playgrounds Prev Med 2009 48 140 143 10.1016/j.ypmed.2008.11.005 19063915

[B45] Roemmich JN Epstein LH Raja S Yin L The neighborhood and home environments: disparate relationships with physical activity and sedentary behaviors in youth Ann Behav Med 2007 33 29 38 10.1207/s15324796abm3301_4 17291168

