# Supplementary material for: Measuring the neighborhood environment: associations with young girls' energy intake and expenditure in a cross-sectional study
Source: Int J Behav Nutr Phys Act. 2010 Jun 1;7:52. doi: 10.1186/1479-5868-7-52 (PMC2893449; doi:10.1186/1479-5868-7-52)
Supplement: Additional file 1 — Neighborhood Audit Tool Items, Standardized Regression Coefficients, and Raw Cronbach's Alphas for Scales Derived From Exploratory Factor Analysis. This file describes the results from exploratory factor analysis, including the individual neighborhood audit tool items, standardized regression coefficients, and raw Cronbach's alphas for each of the five derived scales. [file 1479-5868-7-52-S1.DOC]

**Neighborhood Audit Tool Items, Standardized Regression Coefficients and Raw Cronbach’s Alphas for Scale**s Derived From Exploratory Factor Analysis (n = 215)

| | Item #. Description | Mixed residential and commercial | Food and retail | Recreation | Walkability | Physical disorder | | --- | --- | --- | --- | --- | --- | | 2b. Two-, three-, four-, five-, or six-family home ("walk-ups") | 0.84 |  |  |  |  | | 2c. Apartment building/ complex or condominium | 0.79 |  |  |  |  | | 12a. Speed bump/ speed hump/ raised crosswalk | 0.71 |  |  |  |  | | 4c. Place of worship (e.g. church, synagogue, convent) | 0.70 |  |  |  |  | | 3f. Convenience or small grocery store | 0.69 |  |  |  |  | | 2d. Apartment over retail in multi-story building | 0.63 |  |  |  |  | | 18a. Whole or broken bottles or cans visible in streets, yards, or alleys | 0.63 |  |  |  |  | | 3m. Warehouses, factories or industrial buildings | 0.60 |  |  |  |  | | 6c. Abandoned building or vacant lot | 0.58 |  |  |  |  | | 4i. Community center | 0.52 |  |  |  |  | | 3i. Bar or liquor store | 0.51 |  |  |  |  | | 3a. Gas station | 0.49 |  |  |  |  | | 4d. Day care or preschool | 0.49 |  |  |  |  | | 7a. Presence of sidewalks | 0.46 |  |  |  |  | | 3k. Pharmacy or drug store |  | 0.96 |  |  |  | | 3h. Strip mall or shopping center |  | 0.81 |  |  |  | | 3b. Chain fast food restaurant |  | 0.72 |  |  |  | | 3e. Supermarket |  | 0.71 |  |  |  | | 3c. Other convenience food restaurant |  | 0.68 |  |  |  | | 3j. Laundry or dry cleaners |  | 0.68 |  |  |  | | 3d. Full-service restaurant |  | 0.66 |  |  |  | | 3g. Coffee shop |  | 0.63 |  |  | 0.61 | | 13. Block has crossing aids for pedestrians and bicyclists to cross the street safely |  | 0.58 |  |  |  | | 1. Both residential and non-residential land uses visible in block |  | 0.57 |  |  |  | | 4j. Health care offices, clinic or hospital |  | 0.45 |  |  |  | | 5b. Park |  |  | 0.92 |  |  | | 5h. Walking or hiking trails |  |  | 0.77 |  |  | | 5f. Sports/ playing field, basketball court or tennis court |  |  | 0.62 |  |  | | 5c. Playground, excluding school playgrounds |  |  | 0.55 |  |  | | 16b. "Complete" sports equipment (i.e. all necessary elements are visible) |  |  | 0.53 | 0.49 |  | | 7c. Street shoulders or wide outside lanes |  |  |  | 0.98 |  | | 12c. Curb bulb out/ curb extension |  |  |  | 0.64 |  | | 12d. Traffic circle/ roundabout |  |  |  | 0.59 |  | | 15. Block has street signs that ask drivers to watch out for children and/or pedestrians |  |  |  | 0.53 |  | | 16a. Playground equipment, including school yards |  |  |  | 0.46 |  | | 18b. Garbage, litter or broken glass in the street or on the sidewalks |  |  |  |  | 0.87 | | 4k. Other government buildings (e.g. social security, social services, employment office, etc.) | |  |  |  | 0.69 | | 18c. Graffiti on the buildings, signs or walls |  |  |  |  | 0.67 | | 6d. Railroad tracks (functioning), bridge, tunnel, highway or overpass |  |  |  |  | 0.47 | | Cronbach's alpha | 0.87 | 0.83 | 0.76 | 0.50 | 0.58 | |
| --- | --- | --- | --- | --- | --- | --- | --- | --- | --- | --- | --- | --- | --- | --- | --- | --- | --- | --- | --- | --- | --- | --- | --- | --- | --- | --- | --- | --- | --- | --- | --- | --- | --- | --- | --- | --- | --- | --- | --- | --- | --- | --- | --- | --- | --- | --- | --- | --- | --- | --- | --- | --- | --- | --- | --- | --- | --- | --- | --- | --- | --- | --- | --- | --- | --- | --- | --- | --- | --- | --- | --- | --- | --- | --- | --- | --- | --- | --- | --- | --- | --- | --- | --- | --- | --- | --- | --- | --- | --- | --- | --- | --- | --- | --- | --- | --- | --- | --- | --- | --- | --- | --- | --- | --- | --- | --- | --- | --- | --- | --- | --- | --- | --- | --- | --- | --- | --- | --- | --- | --- | --- | --- | --- | --- | --- | --- | --- | --- | --- | --- | --- | --- | --- | --- | --- | --- | --- | --- | --- | --- | --- | --- | --- | --- | --- | --- | --- | --- | --- | --- | --- | --- | --- | --- | --- | --- | --- | --- | --- | --- | --- | --- | --- | --- | --- | --- | --- | --- | --- | --- | --- | --- | --- | --- | --- | --- | --- | --- | --- | --- | --- | --- | --- | --- | --- | --- | --- | --- | --- | --- | --- | --- | --- | --- | --- | --- | --- | --- | --- | --- | --- | --- | --- | --- | --- | --- | --- | --- | --- | --- | --- | --- | --- | --- | --- | --- | --- | --- | --- | --- | --- | --- | --- | --- | --- | --- | --- | --- | --- | --- | --- | --- | --- | --- | --- | --- | --- | --- | --- | --- | --- | --- | --- | --- | --- | --- |
